# Recommendations for school-going students post CoVid-19 in Bangladesh

**DOI:** 10.6026/97320630017500

**Published:** 2021-04-30

**Authors:** Humayun Kabir, Md. Kamrul Hasan, Mohammad Toyabur Rahaman Bhuya

**Affiliations:** 1 Department of Public Health, North South University, Dhaka, Bangladesh; 2 Department of Biochemistry and Molecular Biology, Tejgaon College, National University, Gazipur - 1704, Bangladesh; 3 CRP Nursing College, Savar, Dhaka - 1343, Bangladesh; 4 Diabetic Association Nursing Institute, Narayanganj, Bangladesh

**Keywords:** CoVid-19, SARS-CoV-2, school closure, mental health of children, policymaking, reopening school

## Abstract

The CoVid-19 pandemic caused by SARS-CoV-2 has taken more lives than any other pandemic so far, with non-pharmacological interventions such as lockdown, school closures, and travel bans, especially social distance, abounding around the world. With limited resources, these interventions pose the ultimate challenge to the education system in developing countries like Bangladesh, especially in providing uninterrupted education for all children in rural areas, where a significant number of students are enrolled in this area. However, the initiative to close schools for an extended period has affected children physically, emotionally, socially, and in various ways. Noteworthy, it demands to reopen to protect the future of children. Schools have reopened in many countries around the world. It is of interest to document recommendations for school-going students post CoVid-19 in Bangladesh using evidence-based data, information, and knowledge. We document such data in the context of Bangladesh to take such policy initiatives.

## Background

The ongoing pandemic of the 2019 novel coronavirus, known as severe acute respiratory syndrome coronavirus 2 (SARS-CoV-2), was first reported in late December 2019 in Wuhan, China [1,2]. As of November 23, 2020, more than 100,805,909 positive cases and 2,164,969 death due to CoVid-19 have been reported in 218 countries and territories [3]. Bangladesh reported the first case of CoVid-19 on March 8, 2020 [4] then after the curve started mounting on November 23, 2020, the total number of cases reported was 447341, and the number of deaths was 6388 [5].

The government announced the closure of every educational institution on March 18, 2020, emphasizing physical distance to lessen the outspread of the virus, suspending the face-to-face education in the classrooms of 42 million students, although some of them started online classes for the first time in Bangladesh [6]. However, evidence suggests that online learning activities are being impeded by several hindrances, such as lack of advanced technology, internet expenses, and extra time for parents and teachers [7].

Approximately, there are 21.6 million primary and 13 million secondary school-going students in Bangladesh, where 76% of secondary schools and roughly 60% of primary schools are positioned in the rural area [8]. Most of the school-going children of Bangladesh, especially in the rural areas have poor online class infrastructures. According to BTRC (Bangladesh Telecommunication Regulatory Commission), out of 160 million people in Bangladesh, unfortunately, 100 million can access the internet, where 95.16 million people have to get internet access through mobile phone [9]. Only 5.6% household affords computer (desktop or laptop) [10] and about 54 % and 59% of rural households don't have any internet access and smart phone access [11]. The other constraints regarding online class conduction include lack of competent and experienced teachers, access to necessary gadgets, electricity [12], etc. resulting in a highly skewed interactive class between urban versus rural schools, poor versus rich around the country [12],[13]. Besides, due to prolonged campus closures, the government has commenced broadcasting primary and secondary school classes on the national TV channel, keeping in mind that family-level TV sets are available for these families [14]. However, it is estimated that more than 23 million households do not have access to TV and TV is a popular recreation centre for households [15] which has become a source of education nowadays. The efficacy of the practice of closing schools is curtailed by the exposure of the non-school community, and approximately 70% of the students are affected by it [16]. However, it is of interest to document recommendations for school-going students post CoVid-19 in Bangladesh using evidence-based data.

## Current Lockdown scenario in Bangladesh:

Lockdown-based prevention stratagems have led to laxity among the general population in Bangladesh as the earnings of the general people are affected [17][18]. Hence, the government may be willing to proceed without further lockdown strictly despite the second wave of CoVid-19 [19][20] where high priority will be given to the ongoing economy [21]. Nevertheless, the World Bank estimates that Bangladesh could reduce its Gross Domestic Product (GDP) by 1.6% during a global economic crisis due to pandemic, alongside, the Bangladesh Bureau of Statistics (BBS) reporting 5.24% growth [22]. However, with the emphasis on wearing masks, there may have no new strategies to maintain a nationwide lockdown for further spikes [23]. Every citizen in Bangladesh is required to wear a mask, but a large number of people step outside without personal protection [24]. Thus, the effectiveness of prolonging school closure is in question. School-related contact for children may be diminished for them whereas non-schooling social transmission is out of control [25].

## Lockdown, online education, and the consequences

Schools confer the highest precedence to meet the mental health-related needs of the child [26]. Protracted distance from friends and teachers, counsellors, trainers, relatives, or family members is detrimental to the mental health of children [27]. This can drag them emotionally vulnerable, altering their mental adjustment system leading to traumatic experiences [28]. Postponing exams owing to the pandemic leads students to more stress [29]. School suspension has possessed problems such as fear, crisis, stress, etc. [30]. In the review study, high-level of anxiety, depression, and traumatic symptoms were reported among the children during this period [31]. A study reported about 58% of parents were asking for psychiatric care for their children during the pandemic as the children were detained from their routine and conventional classes for a longer period [32].

A survey found that 69.3% of parents were distraught about the increase in their children's internet use during this pandemic [33]. School-aged children spend more time on social media, increasing the risk of using problematic internet, smart phone applications, and social media [30]. As well, lockdown increased online gaming time [34]. However, children were at home for an extended period, and thus gaming addiction shot up [35]. Therefore, lockdown indirectly increased the online gaming period among child gamers [34]. Addiction born, as a result of excessive internet use, can accentuate dependence in children [36]. Moreover, economic devastation and increased unemployment rates of parents indirectly affect child abuse. According to the Bangladesh Police Headquarters, the rate of child abuse during the pandemic augmented in multiple folds, with about 680 cases of child abuse occurring between January and June, up from 206 in June [37][38].

The lockdown led to domestic violence, where a study argued that extended detention of children at home in low-income families could lead to potential social crises and inequalities [39][40]. In general, 6.1% of the population in Bangladesh has developed suicidal behaviour due to CoVid-19 [41]. However, CoVid-19 related suicidal ideation and depression were reported by 5% and 33.3% of the Bangladeshi population respectively, with the younger age group at greater risk [42]. Lockdown made students desolate and might upsurge the potential risk of suicide [43].

Apart from academic services, school plays a significant role in keeping children physically and mentally sound and controlling their obesity. Additionally, some schools provide food and shelter as well [44]. The school closure strategies affected the nutritional status of the students, as they didn't get free food from the school [45]. Lockdown also lessened the playing time, resulting in increased body weight of children [46][47]. Behaviour problems turning out to be a serious issue in children due to reduction of physical exercise during school closures were addressed [48]. However, prolonged lockdown can interfere with physical activity, change diet, increase weight, and change their lifestyle and sleeping pattern [33][46].

## Recommendations to mitigate current conditions

We recommend the following strategies in the context of CoVid-19 to students.

## 1. Following CDC guidelines for reopening

Follow the CDC (Centres for Disease Control and Prevention) guidelines for partial withdrawal of the School Closure Strategy. Along with, allow students, teachers, and staff to cooperate in shifts in each of the alternate sections and broadcast a live stream and record of the class for the rest and those who are not coming [50] [51].

## 2. Mitigating the financial and nutritional impact

The economic resistance posed by CoVid-19 should be considered, and financial support or funding for children living in poverty should be taken over precedence. All nutrition-related programs should be re-launched to address malnutrition in children, including the equitable distribution of food to each student [39].

## 3. Introducing campus-based prevention strategies

Campus-based prevention strategies can be implemented by ensuring mask-wearing, hygiene measure, temperature checking, adequate sanitation, and especially physical distancing as well as avoiding some activities like school games, sports, assemblies, and other mass gathering performance [50][52][53][54]. Telehealth should be available at the school level [55] to scrutinize suspected and confirmed CoVid-19 cases for ensuring self-isolation and to develop screening capabilities for suspected students [51]. Necessary actions should be taken by local communities based on contact tracing data and situational analysis [50][53].

## 4. Ensuring/Developing campus-based preventive strategies

Strict campus-based infection prevention strategies can be enforced to certain activities such as school games, sports, assemblies, and other public performances, in addition to ensuring masks, hygienic measurements, temperature verification, adequate sanitation, and especially physical distancing [52], [53]. As well as e-health should be available at the school level. Even in the first hour of class, teachers can confer in child-friendly language, the preventive strategies to enhance their knowledge that may enrich the preventive practices of their family members, where a large number of Bangladeshis are illiterate (about 25.3%) [56], may not be able to access health education usually.

## 5. Assessing campus-based preventive strategies 

Public health experts, health professionals, counselors, and social workers should visit the school on a priority basis to assess preventive practices as well as assessed the health status [40]. Along with screening and evaluating the health status of the students just after reopening especially those who were vulnerable during the CoVid-19 pandemic [40]. Given the children, space to express their health and illness and their family's feelings should be taken into account. However, when the medical services for students are urgently needed, school authorities can coordinate closely with families to liaise with local practitioners and medical specialists.

## Conclusion:

We document recommendations for school-going students post CoVid-19 in Bangladesh using evidence-based data, information, and knowledge as available elsewhere in Norway and other developed nations [52][53]. However, the decision to reopen the school is based on the drawbacks of distance learning, already discussed above. Of all the decisions that have not yet been made in Bangladesh, where China started inaugurating the post-CoVid-19 school opening strategies [57]. Also, the European countries are reinstating the face-to-face schooling system [58]. Henceforth, we support the reopening of the schools based on the evidence of children's educational, physical, mental, social, and emotional well-being. We also suggest more research for establishing universal education strategies than just shutting down schools during a health crisis.

## Figures and Tables

**Figure 1:**
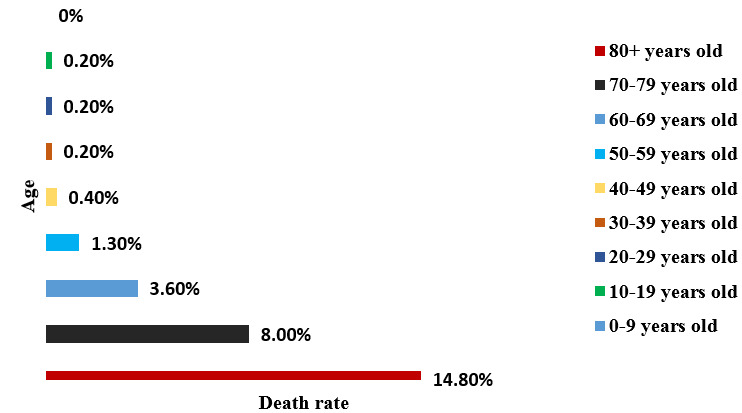
Comparison between age range and death rate [49]

## References

[1] Hossain A (2021). EClinical Medicine..

[2] Pollan M (2020). Lancet..

[3] https://www.worldometers.

[4] https://www.garda.com.

[5] https://corona.gov.bd.

[6] https://iiste.org.

[7] https://www.sysrevpharm.org.

[8] https://www.dw.com.

[9] https://www.universityworldnews.com.

[10] https://www.newagebd.net.

[11] https://www.thefinancialexpress.com.bd.

[12] https://www.thefinancialexpress.com.bd.

[13] https://www.unicef.org.

[14] http://blog.brac.net.

[15] https://network.aljazeera.net.

[16] Viner RM (2020). Lancet Child Adolesc Health..

[17] Biswas RK (2020). Int J Health Policy Manag..

[18] https://www.newindianexpress.com.

[19] https://www.aa.com..

[20] https://bdnews24.com.

[21] https://tbsnews.net.

[22] https://tbsnews.net.

[23] http://www.xinhuanet.com.

[24] https://www.thefinancialexpress.com.bd.

[25] https://www.theguardian.com.

[26] Atkins MS (2010). Adm Policy Ment Health..

[27] https://onlinelibrary.wiley.com.

[28] Joseph SJ (2020). Psychiatr Danub..

[29] Chen Q (2020). Asian J Psychiatr..

[30] Chen IH (2020). J Am Acad Child Adolesc Psychiatry..

[31] de Miranda DM (2020). Int J Disaster Risk Reduct..

[32] Patra S (2020). Asian J Psychiatr..

[33] Adibelli D, Sumen A (2020). Child Youth Serv Rev..

[34] http://www.ijpronline.com.

[35] Poletti M, Raballo A (2020). Euro Surveill..

[36] Deslandes SF, Coutinho T (2020). Cien Saude Colet..

[37] https://onlinelibrary.wiley.com.

[38] https://www.thefinancialexpress.com.bd.

[39] Van Lancker W, Parolin Z (2020). Lancet Public Health..

[40] Donohue JM, Miller E (2020). JAMA.

[41] Mamun MA (2020). Heliyon.

[42] Mamun MA (2020). J Affect Disord..

[43] Mamun MA (2020). Asian J Psychiatr..

[44] de Araujo LA (2021). J Pediatr.

[45] Sheikh A (2020). J Glob Health..

[46] Pietrobelli A (2020). obesity..

[47] Rundle AG (2020). Obesity..

[48] Liu Q (2021). J Affect Disord..

[49] https://www.worldometers.

[50] https://www.cdc.gov.

[51] Johansen TB (2020). Euro Surveill..

[52] Armitage R, Nellums LB (2020). Lancet Glob Health..

[53] Fantini MP (2020). Ital J Pediatr.

[54] Heavey L (2020). Euro Surveill.

[55] Reynolds CA, Maughan ED (2015). J Sch Nur.

[56] https://data.worldbank.org.

[57] https://www.bbc.com.

[58] https://www.bbc.com.

